# Association of Gangrene and Tetanus

**Published:** 1891-01-31

**Authors:** 


### ASSOCIATION OF GANGRENE AND TETANUS.

The frequent occurrence of gangrenous septicæmia and of tetanus after wounds, especially compound and comminuted fractures to which dirt and earth have gained access, is a fact well known to surgeons, while the presence in the outer world, and generally in cultivated soil, of the septic vibrio of Pasteur and the bacillus of tetanus of Nicolaier is established by numerous observations and experiments. But while it has been found in experiments on animals that inoculations with garden soil are followed by septicæmia within a few days, or even hours, or by tetanus after four, five, or more days, the incubation period of tetanus being the longer, the two diseases have never been observed consecutively in the same individual, as they are not infrequently in man. Prof. Verneuil, in *La Semaine Médicale*, discusses this seeming discrepancy between experimental and clinical experience, and he accounts for it by the greater fatality of gangrenous septicæmia in the lower animals, especially under expectant, *i.e.*, no treatment, death supervening before the tetanus has had time to develop itself. It is only when the bacillus of tetanus happens to exist alone that it has an opportunity for producing its effects. In man the incubation periods are longer, though in the same proportion; and when the progress of the septic disease has been arrested by appropriate local treatment, or by amputation of the gangrenous member, the tetanus bacilli having already entered the circulation and gained access to the nerve centres, tetanus follows in due course, so that the two diseases may coexist or appear successively in man though they never do so in other animals. In such cases the septicæmia always precedes the tetanus; not, however, as cause and effect, as was thought before the discoveries of Nicolaier and others, but because the inoculation of the two took place simultaneously, and the latter has the longer period of incubation. Two years ago Professor Verneuil published in the *Révue de Chirurgie* several illustrative cases; and now in the paper before us he gives three others. One was that of a cavalry soldier who sustained a compound comminuted fracture of the forearm, into which some of the dust and earth of the riding school entered. Hopes were at first entertained that the limb might be saved, but on the third day well marked symptoms of gangrenous septicæmia set in, though they subsided after amputation had been performed. Four days later, or seven days from the accident, tetanus appeared, and in spite of all treatment, proved fatal on the twenty-second day, when the wound of the amputation was completely healed. The other two cases occurred in the practi ce of Professor Tédenat, of Montpellier. One was that of a man who had a compound dislocation of the wrist, also caused by a fall from a horse. Earth and manure in this case had penetrated the wound. Rigors appeared in a few hours, gangrene next day, and tetanus on the seventh day, proving fatal in forty-eight hours. The last was a girl who had sustained a compound dislocation of the ankle joint, the lower end of the tibia being encrusted with dirt from the floor of a slaughter-house. On the third day there was superficial gangrenous inflammation extending to the knee, which subsided after multiple incisions; on the twentieth day tetanus supervened, but she ultimately recovered, and later on resection of the ankle was followed by satisfactory restoration of the limb.

